# Sudden Cardiac Death and Ex-Situ Post-Mortem Cardiac Magnetic Resonance Imaging: A Morphological Study Based on Diagnostic Correlation Methodology

**DOI:** 10.3390/diagnostics12010218

**Published:** 2022-01-17

**Authors:** Giuseppe Bertozzi, Francesco Pio Cafarelli, Michela Ferrara, Nicola Di Fazio, Giuseppe Guglielmi, Luigi Cipolloni, Federico Manetti, Raffaele La Russa, Vittorio Fineschi

**Affiliations:** 1 Department of Clinical and Experimental Medicine, University of Foggia, 71100 Foggia, Italy; giuseppe.bertozzi@unifg.it (G.B.); cafarellifrancesco@libero.it (F.P.C.); giuseppe.guglielmi@unifg.it (G.G.); luigi.cipolloni@unifg.it (L.C.); raffaele.larussa@unifg.it (R.L.R.); 2 Department of Anatomical, Histological, Forensic and Orthopedic Sciences, Sapienza University of Rome, 00185 Rome, Italy; michelaferrara13@gmail.com (M.F.); nicola.difazio@uniroma1.it (N.D.F.); federico.manetti@uniroma1.it (F.M.)

**Keywords:** PMMRI, sudden death, post-mortem imaging

## Abstract

During the last years, post-mortem imaging has gradually been assumed within research in the field of forensic pathology. This role appears to be clearly and simply applied in the trauma field with the use of conventional radiography or Post Mortem Computed Tomography (PMCT). Recently, particular attention was paid to cardiovascular imaging using Post Mortem Magnetic Resonance Imaging (PMMRI). The present experimental study aims to: (i) confirm the efficacy of a Post Mortem Cardiac Resonance Imaging (PMCRI) study protocol for the study of human hearts collected during the autopsy; (ii) apply the defined protocol on subjects who died of “SCD (sudden cardiac death)”, to identify alterations that could guide subsequent sampling. Two hearts of healthy subjects (A: male 22 years; B: female 26 years), who died from causes other than SCD were collected and compared to hearts that belonged to SCD individuals (C: male, 47 years old; D: female, 44 years old; E: male; 72 years old). The exams were performed on a 1.5 T scanner (Philips Intera Achieva, Best, the Netherlands) on hearts collected during autopsy and after a 30-day formalin fixation. Two readers analyzed the obtained images blindly and after randomization. From the comparison between the data from imaging and the macroscopic and histological investigations carried out, the present study proved the effectiveness of a PMMRI protocol to study ex-situ hearts. Moreover, it suggested the following semeiology in post-mortem SCD cases: the hyperintense area with indistinct margins in the Short Tau Inversion Recovery (STIR) sequence was linked to edema or area of pathological fibers, whereas the hypointense area in the T2-FFE sequences was linked to fibrosis. PMMRI can provide a valuable benefit to post-mortem investigations, helping to distinctly improve the success rate of histological sampling and investigations, which remains the gold standard in the diagnosis of sudden death.

## 1. Introduction

It has not been long since Flach et al. exclaimed “Times have changed!” [1], to underline the clear role that post-mortem imaging was gradually assuming within the forensic pathology field of research. Nowadays, indeed, it has been assuming the role of an autonomous and independent branch as forensic radiology. Parallelly to the development of technologies in the clinical field, forensic pathology has found valid support in the various imaging techniques, also for its highly demonstrative power [2]. This role can be clearly and simply understood in the trauma field (identifying fractures following road traffic accidents, localization of firearm bullets) with the use of conventional radiography or post-mortem computed tomography (PMCT) [3]. Therefore, it is not surprising the growing interest in magnetic resonance and its ability to provide a high qualification of the tissue studied and excellent anatomical details with high-quality imaging, above all to characterize and identify alterations affecting the brain, heart, subcutaneous fat tissue, and abdominal organs [4]. Such is its potential that some authors believe it is the method capable of replacing the traditional autopsy [5].

Among all the areas of interest, cardiovascular imaging plays a central role in the research of PMMRI (Post-Mortem Magnetic Resonance Imaging), although there is no universally shared standardized protocol. Therefore, the present experimental study aims to: (i) confirm the efficacy of a PMMRI study protocol for the study of human hearts collected during the autopsy; (ii) apply the defined protocol on subjects who died of SCD (sudden cardiac death), to identify alterations that could guide subsequent sampling.

## 2. Materials and Methods

### 2.1. Selected Cases

The full characteristics of the selected cases are shown in Table 1.

Two hearts of healthy subjects (A: male 22 years; B: female 26 years) who died from causes other than SCD were collected. The family and personal cardio-pathological history were nil, and no cardiovascular risk factors were known at the time of death.

Subsequently, during the second phase, two hearts that belonged in life to deceased individuals “within one hour of the onset of symptoms in an apparently healthy subject” [6] (C: male, 47 years old; D: female, 44 years old) and a heart of “one whose illness was not so severe as to predict such a sudden outcome” (E: male; 72 years old) were used. Only three cases of witnessed deaths were involved in the study to reduce the variables due to the other definitions of sudden death. As regards the circumstantial data, subject C was a worker in a factory who, during the working hours, had felt sick and, after a few minutes, died in front of his colleagues. The personal medical history was negative for cardiovascular diseases, while the family history showed that the father had died of sudden death at the age of lesser than 65 years old. D concerned an obese subject, with no personal medical history, who felt suddenly sick, causing her sister to contact the local emergency service, which arrived on the spot and noted the death of the woman. Subject E suffered from hypercholesterolemia and hypertension under pharmacological treatment and was hemodynamically well-balanced; following a verbal altercation with a neighbor, he died at home in the presence of his wife.

### 2.2. Technical Heart Preparation and MRI Protocol

The exams were performed on a 1.5 T scanner (Philips Intera Achieva, Best, The Netherlands) on hearts collected during autopsy (performed 24 h after death without the corpses staying in the cold room) and placed in a polymer container with 10% formalin solution (at the same 30-day interval time from the sample collection). The entire disposal was then fitted in the center of a 16-channel TORSO XL coil and placed in the MRI scanner, as in our previous study [7]. Firstly, the material must be MRI-compatible, which means that it does not contain any metallic or ferromagnetic component that could interfere with the magnetic field. Secondly, the entire disposal has to fit in the center of the 16-channel TORSO XL coil and be well stabilized to limit the movement of the artifacts. The heart was placed in the isocenter of the magnet with its long axis aligned parallel with the direction of the static magnetic field in the MRI scanner. 

The exams were performed on a 1.5 T scanner (Philips Intera Achieva, Best, The Netherlands—Release software 3.2.1) equipped with a gradient system enabling 33 mT/m maximum amplitude and a slew rate of 80 mT/m/ms. The examination protocol included the following sequences:

- Balanced Turbo Field Echo (b-TFE) survey: (thickness 10 mm, field of view (FOV) 450 mm, Time of Echo (TE) 1 ms, Time of Repetition (TR) 3 ms);
- Turbo Field Echo (TFE) T1-3D sequence weighted in axial: (thickness 1 mm, FOV 256 mm, NSA 1, TE 3.4 ms, TR 7.4 ms, Time Inversion (TI) 862 ms);
- T1 Spin Echo (SE) Spectral pre-saturation inversion recovery (SPIR) sequence weighted in axial: (thickness 3.5 mm, FOV 230 mm, NSA 2, TE 15 ms, TR 583 ms);
- T2-3D VISTA (Volume Isotropic Turbo-Spin Echo Acquisition) sequence weighted in axial plane: (thickness 2 mm, FOV 230 mm, NSA 1, TE 227 ms, TR 2000 ms);
- Short Tau Inversion Recovery (STIR) sequence weighted in axial plane: (thickness 5 mm, FOV 270 mm, NSA 3, TE 70 ms, TR 4281 ms);
- T2 (Fast Field Echo) FFE in the axial plane (thickness 5 mm, FOV 226 mm, NSA 2, TE 23 ms, TR 620 ms);
- 3D b-FFE in the axial plane (thickness 1 mm, FOV 180 mm, NSA 2, TE 4 ms, TR 7 ms).

3D b-FFE was included in the present protocol to visualize coronary arteries without a contrast agent. Indeed, a coronary angiogram was indirectly obtained with this 3D sequence using the artificial contrast made by coronary filling with formalin solution.

Total examination time lasted less than 40 min. This protocol was elaborated according to the need to study the signal behavior of fixed tissues, investigating mainly T1 and T2 weighted imaging, edema, and calcification. 3D isotropic imaging was preferred to bi-dimensional images due to the possibility of post-processing reconstructions.

### 2.3. Imaging Analysis 

The study of the images was performed blindly (the operators did not know which cases the analyzed image packages belonged to) and after randomization by two readers with experience in cardiac radiology to evaluate the presence/absence of alterations and their localization.

In order to minimize individual variability in the study of cardiac magnetic resonance (CMR) images, it was decided to proceed with the preparation of a structured report for each sample:

1. CMR, with morphological study and tissue characterization;
2. Coronary-MR of coronary arteries, with segmental characterization, according to American Heart Association (AHA) [8,9].

### 2.4. Pathological Evaluation

All hearts were sampled in compliance with the “Guidelines for autopsy investigation of sudden cardiac death” [10] and maximizing sampling as per our previously published study [7]. All the samples thus obtained were treated with standard hematoxylin and eosin (H&E) staining, with trichrome stains (according to Masson, Azan, and Mallory) and PTAH [11,12]. The samples were also examined under a confocal microscope, and a three-dimensional reconstruction was performed (True Confocal Scanner; Leica TCS SPE). 

## 3. Results

### 3.1. Imaging Evaluation

#### 3.1.1. Heart A and B

In cases A and B, both evaluators agreed upon the high technical quality of PM-CMR and coronary imaging. Moreover, no focal, patchy, or diffuse signal alterations were noticed. No significant restrictions of molecular diffusion were detectable in the DWI and ADC maps. Coronary analysis revealed no pathological defect of the lumina, with regular anatomical origin and course. 

#### 3.1.2. Heart C

##### PMCMR

**Figure 1:**
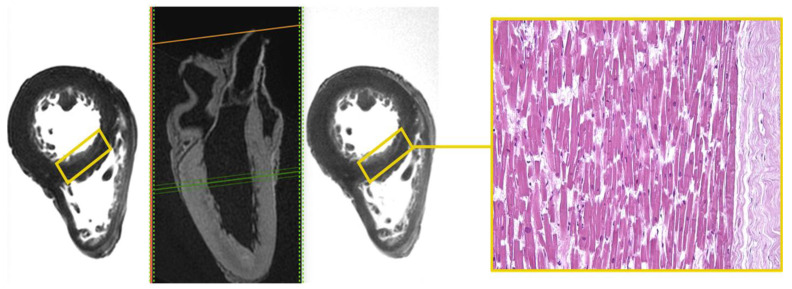
Heart C. STIR—T1-3D—T2 3D sequences. Hyperintense sub-endocardial area in STIR sequence at the mid-cardiac area at the LV septum and the corresponding histological H&E preparation in which foci of myocells characterized by contraction band necrosis, and the presence of amorphous eosinophilic material were visible (×40).

Area of altered signal, with blurred edges and hyperintense in the long TR sequence fluid-sensitive (STIR), was appreciated in the mid-cardiac level, at the sub-endocardial area of the LV septum (Figure 1).

Microscopic analysis demonstrated foci of myocells characterized by sarcorexis, with transverse hypereosinophilic bands and contiguous stretched fibers, separated from each other by the presence of amorphous eosinophilic material.

**Figure 2:**
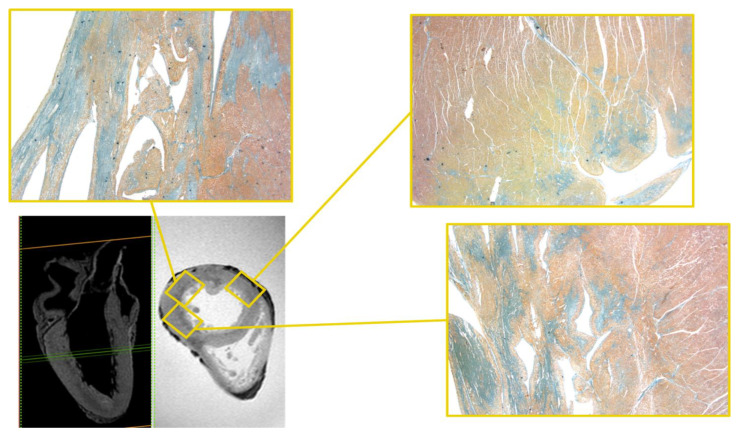
Heart C: T1-3D and T2-FFE sequences. In this latter, a hemi-circumferential in-homogeneously hypointense area of the altered signal the mid-cardiac level, more evident along the LV lateral and posterior wall and the corresponding histological samples stained with Mallory’s trichrome demonstrated extensive areas of fibrosis where they are blue in color (×20, all histological images). Another similar area in the same sequence is appreciated at LV septum-posterior wall angle.

At the same endocardial side of the mid-cardiac level, more evident along the left ventricle (LV) lateral and posterior wall, a hemi-circumferential in-homogeneously hypointense area of the altered signal was highlighted in the T2-weighted and T2-FFE sequences, finding compatibility with extensive areas of fibrotic substitution of myocardial tissue (Figure 2). Another area of a similar altered signal in the same sequences was appreciated at the same level of the LV septum-posterior wall angle. 

There are no clear areas of restriction of diffusivity in the DWI sequences and the corresponding coronary artery disease (CAD) map.

##### Coronary-MR

**Figure 3:**
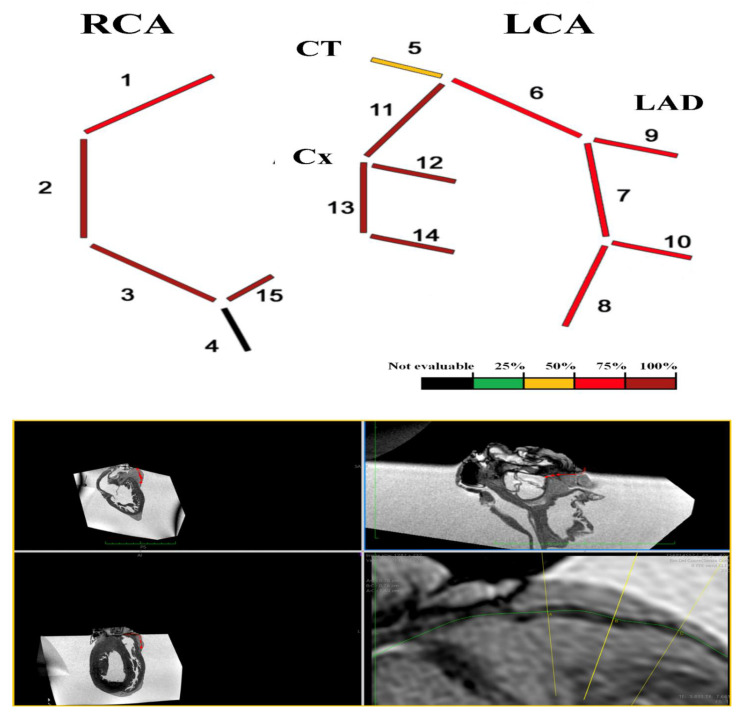
Heart C. Coronary-MR representation according to four-degrees scale [8,9] on the basis of the percentage of cross-sectional area stenosis: 1% to 25%, 26% to 50%, 51% to 75%, and 76% to 100%. Coronary-MR images showing the coronary course and highlighting relative luminal stenosis.

The overall assessment of coronary vessels is shown in Figure 3.

#### 3.1.3. Heart D

##### PMCMR

An abundant and widespread representation of the epicardial adipose tissue. The LV presents an irregular wall thickening, with posterior and lateral walls of greater thickness. The average diameter of the LV was about 11.14 mm.

**Figure 4:**
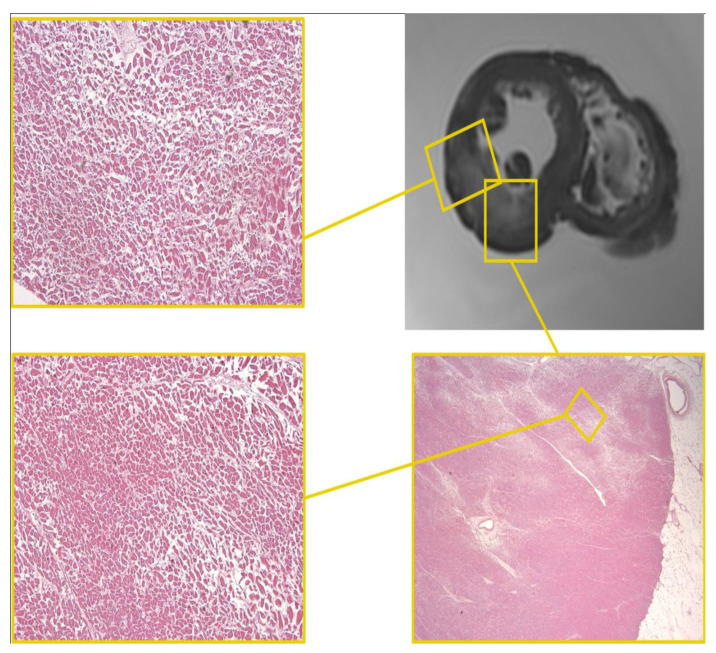
Heart D. STIR—hyper-intense area of altered signal, with blurred edges and sub-endocardial-transmural extension as the mid-cardiac level of the LV posterior and lateral walls and the corresponding histological H&E samples. On routine histological staining (H&E) the areas of altered signal demonstrate the presence of areas of interstitial fibrosis (white areas) (×40).

The large area of the altered signal, with sub-endocardial-transmural extension, blurred edges, and hyper-intense in STIR sequence, was appreciated in the mid-cardiac level of the posterior and lateral LV walls (Figure 4).

**Figure 5:**
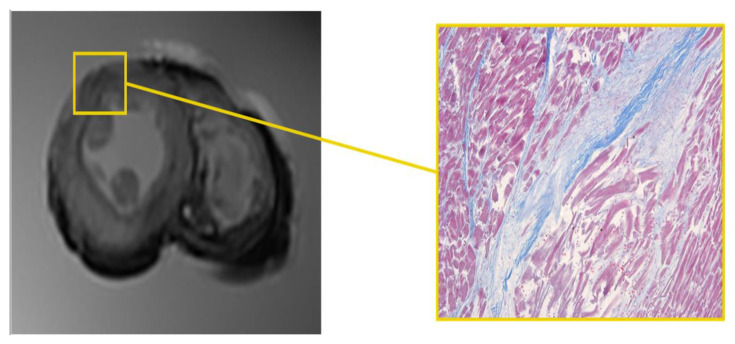
Heart D. T2-FFE. Hemi-circumferential and in-homogeneously hypo-intense area on the epicardial side of the LV anterior wall in the mid-basal area and the corresponding histological samples stained with Mallory’s trichrome (×60). Fibrotic bundles (in blue) cross the muscle bundles, interrupting them.

In the mid-basal area, on the epicardial side, along the anterior wall of the LV, there was an area of altered signal, hemi-circumferential and in-homogeneously hypo-intense in the T2-weighted and T2-FFE sequences. Other areas of similar signal and significance were appreciated, again in the meso-cardiac area, at the septal level, and the postero-septal angle (Figure 5).

No clear areas of diffusivity restriction were appreciated in DWI sequences with high values of band in the corresponding CAD map.

##### Coronary-MR

**Figure 6:**
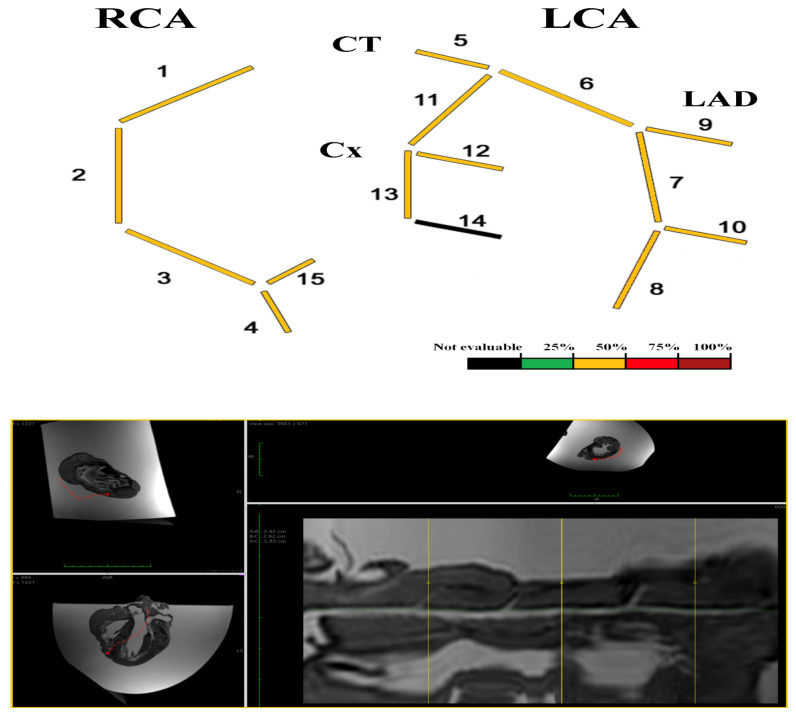
Heart D. Coronary-MR representation according to four-degrees scale [8,9] on the basis of the percentage of cross-sectional area stenosis: 1% to 25%, 26% to 50%, 51% to 75%, and 76% to 100%. Coronary-MR images showing the coronary course and highlighting relative luminal stenosis.

The overall assessment of coronary vessels is shown in Figure 6.

#### 3.1.4. Heart E

##### PMCMR

Besides abundant epicardial adipose tissue, the heart appeared globally hypertrophic, with an average LV diameter of about 18.35 mm. The RV also looked thickened. 

**Figure 7:**
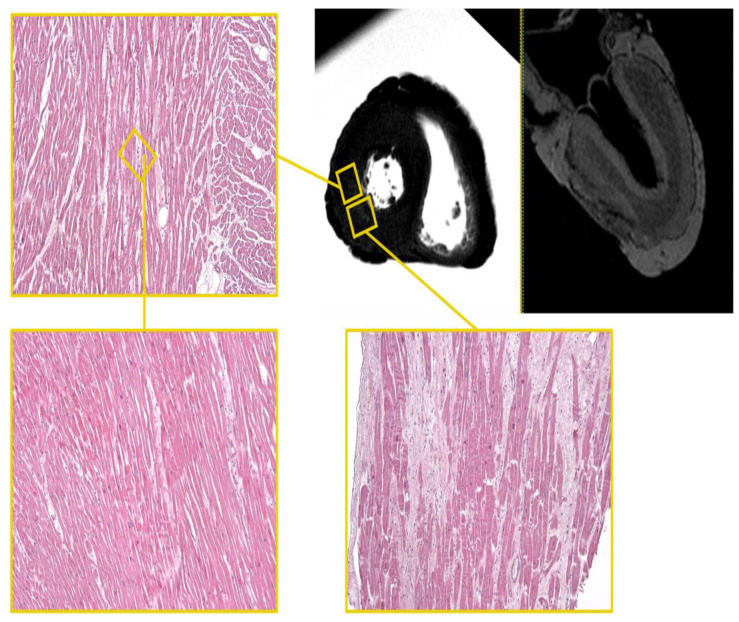
Heart E. STIR—T1-3D sequences. Hyperintense area in the STIR sequence with indistinct margins in the mid-cardiac area at the sub-endocardial level of the LV posterolateral wall and the corresponding histological H&E samples (×60). Foci of myocells characterized by sarcorexis, with transverse hypereosinophilic bands and contiguous stretched fibers, separated from each other by the presence of amorphous eosinophilic material is present in the interstitial space together with predominantly leucocytic cells. Areas of patchy fibrosis affect the left ventricular wall (white areas).

An area of altered signal, with indistinct margins and hyperintense in the STIR sequence, was appreciated in the mid-cardiac area at the sub-endocardial level of the LV postero-lateral site, referable to clinical edema/hypercellularity (Figure 7). Another large area of analogous altered signal, in the same sequence, was evident at the level of the middle third of the septum.

In the T2-FFE sequences, a global reduction of the myocardial signal was documented; this in-homogeneously hypointense finding was more accentuated at the level of the LV lateral and posterior walls in the mid-basal cardiac region (Figure 7).

There were no clear areas of restriction of diffusivity in the DWI sequences and the corresponding ACD map. No clear areas of diffusivity restriction were appreciated in DWI sequences with high values of band in the corresponding ACD map.

##### Coronary-MR

**Figure 8:**
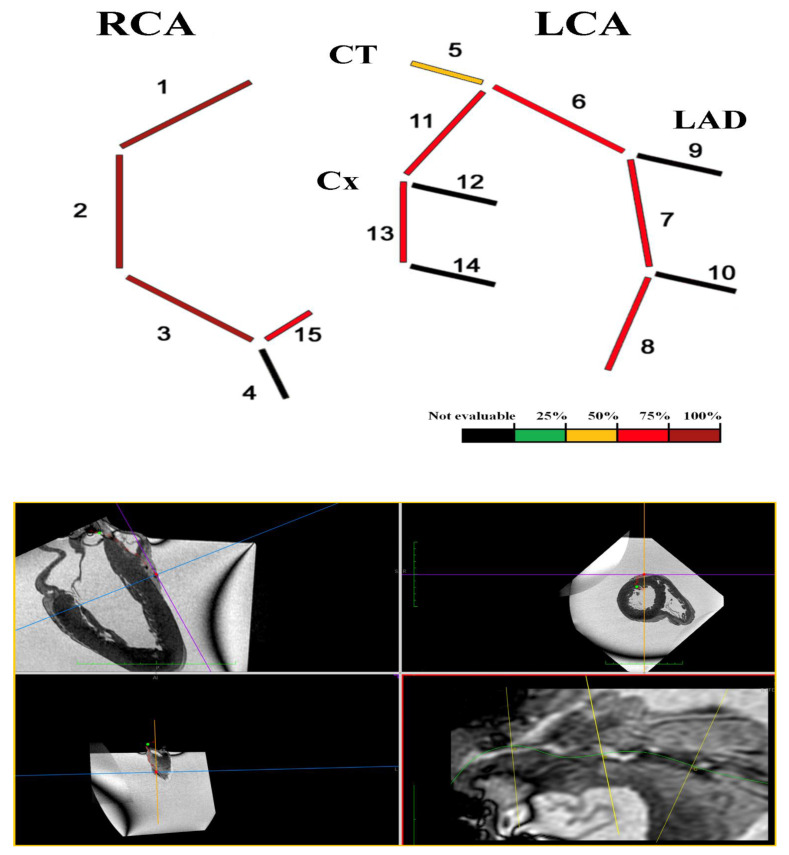
Heart E. Coronary-MR representation according to four-degrees scale [8,9] on the basis of the percentage of cross-sectional area stenosis: 1% to 25%, 26% to 50%, 51% to 75%, and 76% to 100%. Coronary-MR images showing the coronary course and highlighting relative luminal stenosis.

The overall assessment of coronary vessels is shown in Figure 8.

### 3.2. Pathological Evaluation

#### 3.2.1. Heart A and B

Heart and coronary samples taken from cases A and B did not show pathological results, apart from the presence of autolysis of myocells in some fields.

#### 3.2.2. Heart C

**Figure 9:**
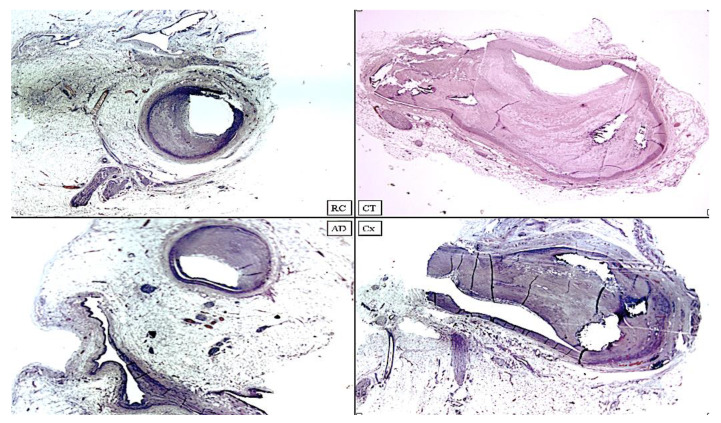
Heart C. Coronary samples in H&E staining. RC: Right Coronary; CT: Common Trunk; AD: Anterior Descending; Cx: Circumflex. Different degrees of reduction in vessel caliber are clearly visible with functionally critical stenoses that can lead to acute coronary syndrome or sudden death (H&E, ×80).

The heart measured 11.8 × 12 × 4.5 cm and weighed 460 g, with a regular representation of the subepicardial fat. The study of the coronary arteries (Figure 9) made it possible to detect critical stenosis of all coronary tracts.

After dissection according to the semi-conservative technique, the left ventricular chamber showed: an anterior wall of 1.2 cm; a lateral wall of cm 1.45; a posterior wall of 1.4 cm; a thickness of the interventricular septum equal to 1.2 cm.

There was nothing to report on the endocardium, which appeared translucent. There was also nothing to report to the right ventricular chamber, with walls 0.6 cm thick and regular volume. The atrial chambers appeared to be of regular volume. The atrio-ventricular valves had thickened valvular laces, as well as pulmonary and aortic semilunar valves.

**Figure 10:**
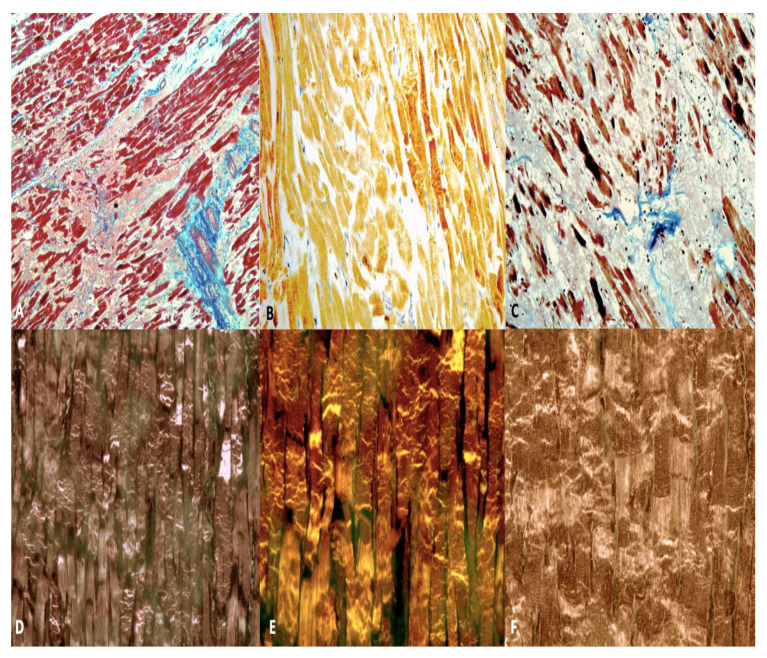
Heart C. The septum shows areas of well-organized fibrosis (in blue) and interstitial edema (in pink) (**A**), bundles of organizing fibrosis are clearly visible (in white) (**C**) (Azan’s Trichrome, ×60). (**B**) Segmentation of the myocardial cells and contraction-band necrosis foci (in brown). (**D**–**F**) Confocal laser microscope: widening of intercalated discs and bundles of contracted myocardium alternating with bundles of distended myocardium with granular disruption of the myocytes were noted in all myocardial sections of LV (PTAH, ×100).

In correspondence with the areas identified on the magnetic resonance examination, it was possible to observe:

- left ventricle: in multiple fields, the myocardial architecture is altered by the presence of extensive areas of fibrotic substitution of the myocardial tissue and by interstitial and perivasal fibrosis that interrupt myocardial continuity, a characteristic mostly represented at the second section and the lateral and posterior walls, above all if compared to the anterior wall at the same section (Figure 10A,C).
- septum: second section, immediately below the endocardium pertaining to the left ventricle, foci of myocells characterized by sarcorexis, with transverse hypereosinophilic bands and contiguous stretched fibers, separated from each other by the presence of amorphous eosinophilic material (Figure 10B); fragmentation of the whole myocell (pancellular lesion) in a pathological band with intense hypereosinophilia of the hypercontracted myocardial cells, extremely short sarcomeres, highly thickened Z lines, and rexis of the myofibrillar apparatus into cross-fiber, anomalous and irregular (Figure 10D,E). Pathological bands were formed by segments of hypercontracted and coagulated sarcomeres.
- The second pattern associated with the previous one was represented by a unique band of 10–20 hypercontracted sarcomeres close to the intercalated disc with a typical aspect of paradiscal lesion (Figure 10F). In this case, the band assumes a dark, dense ultrastructural aspect with very thin Z lines and myofibrils and mitochondria squeezed in the normal portion of the myocyte.

#### 3.2.3. Heart D

**Figure 11:**
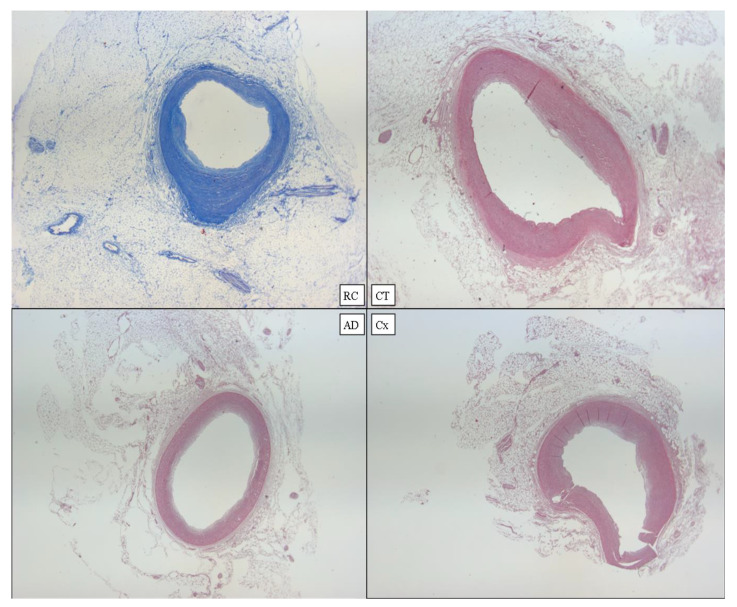
Heart D. Coronary samples: RC with Azan’s staining; CT, AD, and Cx in standard H&E staining (×60).

The heart measured 12 × 11 × 3 cm and weighed 415 g, with an abundant representation of sub-epicardial fat. Absence of alterations in the coronary vessels (Figure 11).

The histological examination showed in the mid-cardiac section of the left ventricle, a polymorphous pathological picture in the heart, characterized by areas of edema and fiber segmentation.

#### 3.2.4. Heart E

**Figure 12:**
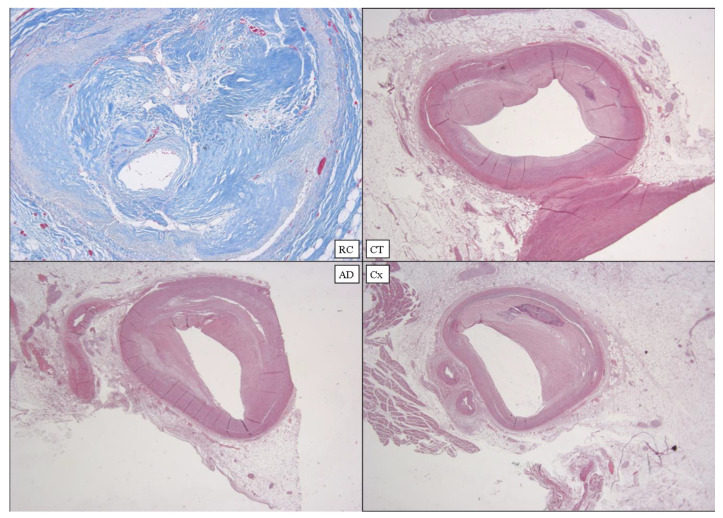
Heart E. Coronary samples: RC with Azan’s staining; CT, AD, and Cx in standard H&E staining (×80).

The heart was of normal shape and size, regular consistency, measured 12 × 11.5 × 7 cm and weighed 550 g, with an abundant representation of subepicardial fat. The coronary study confirmed the PMMRI results (Figure 12).

The macroscopic evaluation of cardiac walls showed an anterior wall equal to 1.5 cm, a lateral wall equal to 1.9 cm, a posterior wall equal to 1.8 cm, and a thickness measured in all correspondence of the interventricular septum equal to 1.6 cm.

At the lateral wall of the left ventricle, the fibers appeared separated by amorphous eosinophilic material, as well as interested by the recurrence of foci of myocardial cells characterized by sarcorexis, with transverse hypereosinophilic bands and contiguous stretched fibers, both in the subepicardial and in the subendocardial area. In particular, the samples were conducted in correspondence with the lateral and posterior walls of the left ventricle. The same findings were found at the middle third of the septum at the same level.

### 3.3. Cause of Death

#### 3.3.1. Case C

The death of C is attributable to sudden cardiac death on an arrhythmic basis, according to the contribution provided by: the circumstantial and clinical-anamnestic data; the autoptic results, which highlighted the presence of three-vassal coronary heart disease with significant stenosis of the coronary lumen; the evidence of myocardial and coronary imaging which identified the presence of suspected areas, subsequently confirmed by the histological examination, and significant stenosis of coronary vessels. In addition, the toxicological investigation excluded causal/co-causal inference from toxic substances (alcohol, drugs) in the death determinism.

#### 3.3.2. Case D

The death of D is attributable to sudden cardiac death on an arrhythmic basis because the integration of all evidence came from: the circumstantial data of death following a sudden pain; the results of the sectoral and macroscopic examination of the heart, which highlighted obesity, with an increase in visceral and subepicardial fat, as well as thickening of the LV lateral and posterior walls, in the absence of significant coronary stenosis; the myocardial and coronary imaging which identified the presence of anomalous areas and widespread irregularity in the context of the myocardial muscle, in the absence of significant coronary stenosis; from the results of histopathological examinations, which allowed us to document a polymorphic pathological picture, characterized by areas of edema and fiber segmentation and confirming the absence of significant coronary stenosis. Even in this case, the toxicological tests resulted negative.

#### 3.3.3. Case E

The death of E is due to sudden cardiac death on an arrhythmic basis, following the analysis of the complete pattern: the circumstantial and clinical-anamnestic data of a hypercholesterolemic and hypertensive subject; the cardiac macroscopic inspection which showed an increase in subepicardial fat, with significant stenosis affecting the three main epicardial vessels; the presence of pathological areas at PMMRI and significant stenosis at coronary-RM; the histopathological examinations, which allowed to confirm the significant coronary stenosis already evident on macroscopic and radiological examination, associated to necrosis with contraction bands of quantitatively significant extent; and the negativity at the toxicological investigation.

## 4. Discussion

The present study had as its objective, first of all, the evaluation of the application of studying the heart following evisceration and, subsequently, proving its “effectiveness” in post-mortem diagnostics in cases of sudden death.

PMMRI, indeed, among the other techniques, is still the least studied and of rare application to forensic pathology, and the first reason is certainly the complexity of its technology [13,14]. In fact, to obtain satisfactory results, it is necessary to adapt the acquisition protocols that must balance the visual field and the resolution obtained (the smaller the visual field, the better the resolution); thus, the examination time required to perform a high-quality PMMRI exam is an important limiting factor. For these reasons, PMMRI is mainly performed in an anatomical region of specific interest and cannot be used as a screening method such as PMCT (Post-Mortem Computed Tomography) [15,16]. On the other hand, the absence of circulation makes it impossible to use dynamic MR clinical sequences.

Nevertheless, its applicability has already been demonstrated in other studies for the diagnosis of hypertrophic cardiomyopathy [17] and myocardial ischemia [18].

In continuity with the previous ones, the present study proposed a protocol for PMMRI that has demonstrated its applicability, with images that were evaluated as being of excellent quality. Furthermore, by the presence of the formolic solution, it was possible to obtain a visualization of the coronaries without having to resort to dynamic sequences.

Potentially, however, in addition to high-contrast resolution and anatomical details, PMMRI could be used to identify specific tissue characteristics, such as in the case of hemorrhage, to establish the temporal evolution of a pathological process. On this basis, PM-CMR is a field of recent growing interest due to its role in distinguishing acute, sub-acute, and chronic infarction, as demonstrated by the studies of Jackowski et al. [19,20]. Swiss authors, in fact, argue that the gold standard in post-mortem diagnostics, the autopsy, is not able to detect the signs of hyperacute infarction, probably due to the short survival times of a few minutes, which would not allow the development of vital reactions within the myocardium, such as edema or necrosis, sufficient to be appreciated. In detail, in very early cases, the results of the macroscopic examination and even of the histological examination may not be able to distinguish the initial ischemic myocardium. In these cases, the autopsy still depends on coronary diagnostics showing severe stenosis or occlusions. The study by Femia et al. [21] also follows a similar line, suggesting, when it is not possible to perform a conventional autopsy, that a high intensity of the T2 signal and an RV/LV ratio can be useful indicators for the diagnosis of both heart attack acute/subacute myocardial and pulmonary embolism.

However, the histological morpho-structural findings typical of cardiopathology do not coincide only with the signs of an “infarct” necrosis, or of myocardial flaccid paralytic tissue with stretching of the necrotic myocardial fibers [22,23,24,25]. In fact, catecholaminic necrosis, or, more precisely, irreversible hypercontraction, can be found, so defined for the extreme reduction of sarcomeres and the very marked thickening of the Z lines, which is never present in normal conditions, with intense hypereosinophilia. This rupture of the myofibrillar apparatus in anomalous transverse bands or pathological bands is also defined as “infarct-like necrosis, microinfarcts, focal myocytolysis, myocytolysis with large bands of contraction, myofibrillar degeneration”. Another type of morpho-functional damage consists in a progressive loss or disappearance of myofibrils with the result of an increasing vacuolation, which begins around the apparently undamaged nucleus. The empty space of myofibrils is filled with small mitochondria and edematous fluid, with the dissolution of the myofibrillar apparatus and loss of the capacity for resynthesis of fibrils. This form of myocellular alteration is specific to myocardial insufficiency of any kind, known as “colliquative necrosis” [26]. Finally, the finding of the “segmentation” of myocardial fibers from rupture of the intercalary discs with myocellular separation, described as a possible preterminal event related to ventricular fibrillation [27].

These different histopathological substrates, however, are not all identifiable recurring to imaging, as far as interpretable with current data. On the other hand, not all the information coming from PMCMR have found, to date, a histopathological equivalent or interpretation, such as diffusion-weighted imaging (DWI) or spectroscopy for the chemical study of tissues [28]. Furthermore, a recent study suggests that formol fixation could induce changes in the MRI signal [29]. Although, therefore, further studies are certainly necessary, compared to PMCT, PMMRI seems to be a very promising method in the study of the myocardium in cases of sudden cardiac death [30,31,32].

## 5. Conclusions

In the present study, although in a few cases examined, the proposed PMCMR-protocol was able to identify areas of altered signal, helping to distinctly improve the success rate of histological sampling and investigation (which remain the gold standard in the diagnosis of sudden cardiac death). Conversely, even if conducted by the guidelines, the pathological areas could not have been sampled, due to a random method. In this regard, another problem raised using PMCMR is the absence, to date, of a codified semeiology to give the correct meaning to the alterations detected with this method in a post-mortem context. From this study, it seems reasonable to link the hyperintense area with indistinct margins in the STIR sequence to edema or the area of pathological fibers and the hypointense area in the T2-FFE sequences to fibrosis. 

These findings offer new perspectives in this field that can integrate with new developments that will occur in the coming years with the main objective of developing shared and standardized protocols.

## Figures and Tables

**Table 1:** Summary table of the main features of cases involved in this study.

Cases	Age	Sex	Autopsy Findings	Heart Measures (CM)	Heart Weight (G)	Maximum Wall Thickness (CM)
A	22	Male	Widespread skull fractures and brain injury from single-shot gunshot	13.2 × 11.4 × 4.5	330	1.3
B	26	Female	Cranial fractures and subdural and subarachnoid haemorrhage from blunt trauma	10.5 × 10 × 3.5	265	1.2
C	47	Male	Diffuse vascular atheromasia	1.8 × 12 × 4.5	460	1.4
D	44	Female	Obesity, diffuse visceral and epicardial fat	12 × 11 × 3	415	1.4
E	72	Male	Diffuse vascular atheromasia	12 × 11.5 × 7	550	1.9

## Data Availability

Not applicable.
